# Outcome evaluation of capacity building and mentorship partnership (CBMP) program on data quality in the public health facilities of Amhara National Regional State, Ethiopia: a quasi-experimental evaluation

**DOI:** 10.1186/s12913-021-07063-2

**Published:** 2021-10-05

**Authors:** Melaku Birhanu Alemu, Asmamaw Atnafu, Tsegaye Gebremedhin, Berhanu Fikadie Endehabtu, Moges Asressie, Binyam Tilahun

**Affiliations:** 1 Department of Health Systems and Policy, Institute of Public health, College of Medicine and Health Sciences, University of Gondar, PO. Box – 196, Gondar, Ethiopia; 2 Department of Health Informatics, Institute of Public Health, College of Medicine and Health Sciences, University of Gondar, Gondar, Ethiopia; 3 Amhara National Regional Health Bureau, Amhara, Ethiopia

**Keywords:** CBMP, Effectiveness, Impact, Outcome, Evaluation, Data quality, OECD, Amhara

## Abstract

**Background:**

Capacity Building and Mentorship Partnership (CBMP) is a flagship program designed by the Ethiopian Ministry of Health in collaboration with six local universities to strengthen the national health information system and facilitate evidence-informed decision making through various initiatives. The program was initiated in 2018. This evaluation was aimed to assess the outcome of CBMP on health data quality in the public health facilities of Amhara National Regional State, Ethiopia.

**Methods:**

A matched comparison group evaluation design with a sequential explanatory mixed-method was used to evaluate the outcome of CBMP on data quality. A total of 23 health facilities from the intervention group and 17 comparison health facilities from a randomly selected district were used for this evaluation. The Organization for Economic Cooperation and Development (OECD) evaluation framework with relevance, effectiveness, and impact dimensions was used to measure the program’s outcome using the judgment parameter. The program’s average treatment effect on data quality was estimated using propensity score matching (PSM).

**Results:**

The overall outcome of CBMP was found to be 90.75 %. The mean data quality in the intervention health facility was 89.06 % [95 %CI: 84.23, 93.88], which has a significant mean difference with the comparison health facilities (66.5 % [95 % CI: 57.9–75]). In addition, the CBMP increases the data quality of pilot facilities by 27.75 % points [95 %CI: 17.94, 37.58] on the nearest neighboring matching. The qualitative data also noted that there was a data quality problem in the health facility and CBMP improved the data quality gap among the intervention health facilities.

**Conclusions:**

The outcome of the CBMP was highly satisfactory. The program effectively increased the data quality in the health facilities. Therefore, the finding of this evaluation can be used by policymakers, program implementers, and funding organizations to scale the program at large to improve the overall health data quality for health outcome improvement.

## Background

Effective health service delivery needs quality data and evidence-based decision making. Health data is critical for different purposes, including health sector reviews, planning, program monitoring, quality improvement, and reporting [1]. In addition, accurate and complete data are vital for appropriate evidence-based decision-making on health expenditure, for responding to countries’ specific health needs and measuring the impact of health programs [2]. As a result, it is necessary to have high-quality data in the health sector. However, healthcare is hugely affected by a lack of quality data, such as measurement error as clinicians give priority to care than to data, missing values mostly in emergencies, and human-related mistakes during data entry and analysis [1, 3, 4].

The health sector data are used to describe the health status, morbidity, trends and cause and effect analysis on health problems. In addition, it helps to assess the effectiveness of health interventions. However, the information system in the health sector has limitations regard to data quality [5]. Low data quality results in poor and inefficient utilization and can result in serious errors in decision-making practice [6].

In Ethiopia, the health facilities had discrepancies in their reported and source documents [7, 8]. There is low data quality in the health facility, which needs interventions on the lower level of the healthcare structure to improve Ethiopia’s quality of healthcare data [7, 9, 10]. The proportion of health facilities that had trained staff for data collection and compilation, guidelines on reporting, and routine inspection of the quality of reports were 17 %, 37 %, and 39 %, respectively. The low data quality results from a lack of trained staff on data collection and compilation, resulting in inferior performance in health data quality [7, 11]. It is estimated that half of the health information system (HIS) staff have no data collection and compilation training. As well, there is no written guideline on reporting and routine processes in half of the district healthcare organizations [7].

In the 2015 Ethiopian Health Sector transformation plan, the information revolution is one of the country’s priority agendas to revolutionize how data is collected, analyzed, and used in the health sector through the change in culture, attitude and use of technology [12].

The Ethiopian Ministry of Health, in collaboration with the local universities, designed a HIS capacity building and mentorship partnership (CBMP) program that aims to strengthen the national HIS and facilitate evidence-based decision making through various initiatives [13]. The program is working to increase the data quality and information utilization in the health facilities [12, 13] through capacity building by training, supportive supervision and mentorship. The CBMP in Amhara region is being implemented since May 2017 as a pilot in three zones (East Gojjam, South Wollo, and Central Gondar zones).

**Fig. 1:**
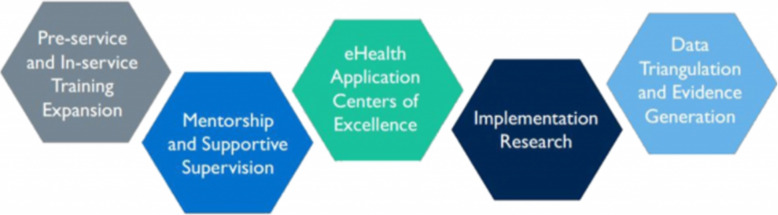
CBMP strategies

The CBMP is a collaborative partnership of six local Universities, the Federal Ministry of Health, and Regional Health Bureaus to achieve the information revolution agenda by creating model health facilities and districts through improvements in data quality and evidence-based decision-making. The project was launched in May 2017 to be implemented for five years [13] with the objectives of (1) improving health data quality and information utilization, (2) improving the capacity of health workers and health managers to prepare, analyze, and use quality health information for evidence-based decision-making, (3) building the capacity of health workers and health managers at all levels to analyze, use, and prepare quality reports, (4) implement district health information system 2 (DHIS 2) at facilities and district health offices and (5) conduct continuous implementation science research to identify what works and what does not in improving data quality and ensuring information use [13]. The project has specific objectives (1) increase the data quality from 28 to 90 % at CBMP site in May 2020, (2) increase the health information system (infrastructure, data quality, and information utilization) to greater than 90 % by May 2020 and strategies (Fig. 1) at the designing stage.

Major strategies.

Even though there is monitoring and assessment in the CBMP, there is a need to conduct the evaluation using a scientific method to have detailed evidence of program effectiveness. Moreover, the relevance of the program to the target population is not well known so far. Therefore, this evaluation was aimed to assess the outcome of CBMP on data quality using the OECD-DAC evaluation criteria (relevance, effectiveness, and impact dimensions) in Amhara National Regional State, Ethiopia.

The evaluation of the CBMP has principal importance in providing information for funders and implementers. The CBMP is a pilot intervention implemented in only three administrative zones (East Gojjam, South Wollo and Central Gondar) in Amhara National Regional State. Therefore, the evaluation result provides valuable information about the relevance, effectiveness, and impact of the CBMP for evidence based decision making. Also, it might help the policymakers to scale up the program to the regional level. Furthermore, the finding can be used as a baseline to quantify the intervention’s cost and benefit analysis for further studies.

## Methods

### Evaluation area and period

**Fig. 2:**
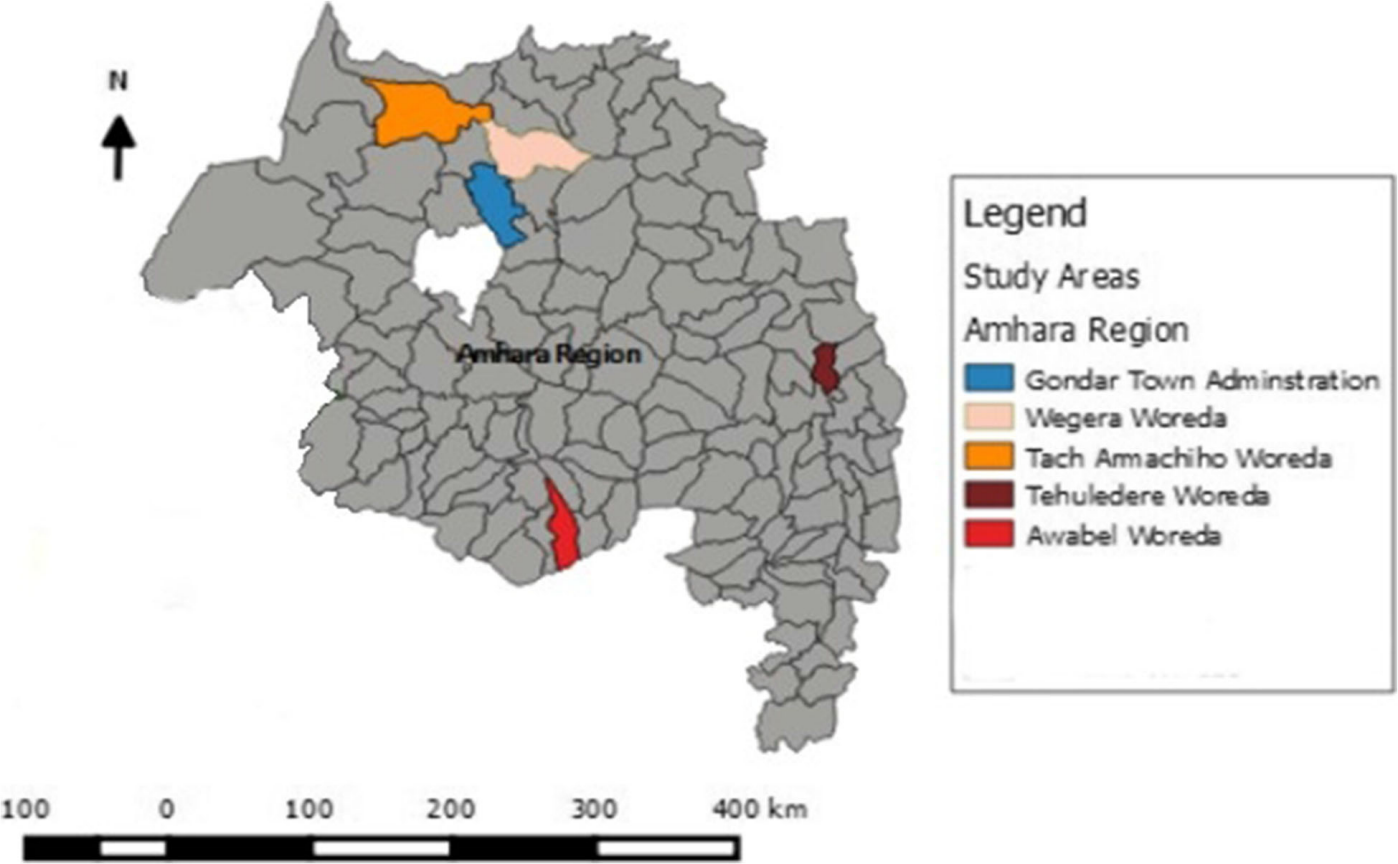
Capacity Building and Mentorship Program implementation sites in Amhara National Regional State, 2018

The evaluation was conducted in East Gojjam, South Wollo, and Central Gondar zones of the Amhara National Regional State, Ethiopia (Fig. 2) [14], from April 28 to June 12, 2020. The evaluability assessment was conducted from July 1 2019, to November 30 2019. The region has a 131.8 person/km^2^ population density and shared a border with Sudan to the west and northwest, Tigray National Regional State to the north, Afar National Regional State to the east, Benishangul-Gumuz National Regional State to the west and southwest Oromia National Reginal State to the south [15]. It has 12 zones, three-city administrations, and 180 districts (139 rural and 41 urban). Moreover, it has 80 hospitals (five referral, two general, and 73 primary hospitals), 847 health centers, and 3,342 health posts [16].

### CBMP intervention description

 The expected effect of the CBMP is to increase the health data quality and information utilization culture for evidence-based decision making on the pilot areas of the Amhara National Regional State, Ethiopia. The program strives to transform the health institutions to the model health institutions.

**Fig. 3:**
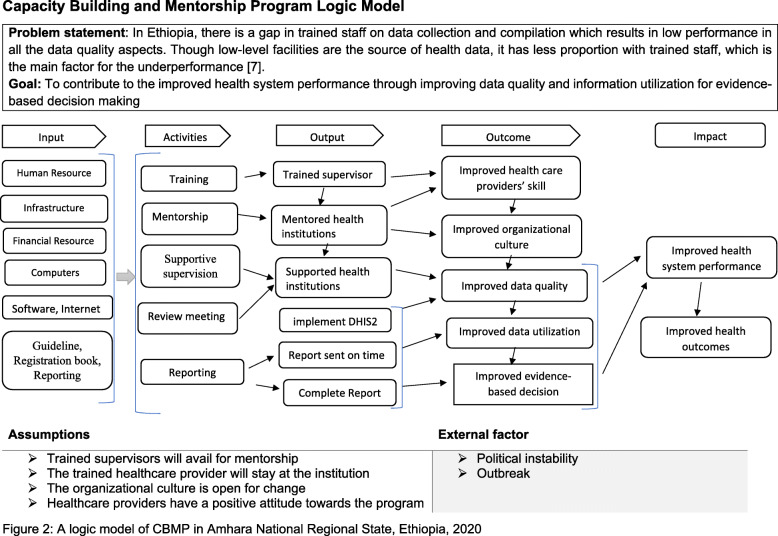
A logic model of CBMP in Amhara National Regional State, Ethiopia, 2020

The CBMP program provides technical support, mentorship and supportive supervision to the health facilities to increase performance on health information system (Fig. 3). The universities are responsible for providing technical support and filling the knowledge gap regarding data quality and information utilization. There are immense resources in the universities to strengthening the health information system in Ethiopia. For instance, there are different specialization training modules in health information, health systems, and policy in the universities, which helps to strengthen the health system. Besides, there are international collaborations at the universities to assist in the implementation of the health information system.

### Evaluation approach and design

A formative evaluation was conducted to evaluate the outcome of CBMP. A counterfactual evaluation approach was employed to assess the impact of the program on data quality.

A matched comparison group evaluation design with a sequential explanatory mixed method was used to evaluate the outcome of CBMP. In addition, the qualitative data complemented the evaluation by assessing the relevance dimension.

The matching comparison evaluation design is a quasi-experimental evaluation design used to estimate the impact of a program. The treatment and comparison groups are typically identified in matched-comparison group designs after the program has already been implemented [17]. Accordingly, health institutions under CBMP were taken as intervention groups, and health facilities that were not under CBMP intervention were considered a comparison group. Thus, the design allows us to estimate the contribution of CBMP on health data quality in the intervention areas.

The average treatment effect was estimated using a propensity score method (PSM) by taking the number of catchment population, the number of catchment health institutions and availability of standard medical record units as a factor to estimate the propensity score. The matching based on the propensity score was done using a stratification matching method. Finally, the average treatment effect was calculated using all the intervention and comparison facilities.

Propensity score matching (PSM) creates a statistical comparison group based on the probability of being in the intervention group by using independent observable characteristics. The matching was done using the propensity score estimated using the Probit model. The average treatment effect was then estimated by the mean difference between the two statistically created groups. There are two assumptions for the effective estimation of PSM. First, unobserved factors do not affect participation (confoundedness). Secondly, there should be an overlap of the propensity score between the participants and nonparticipant samples (common support) [18].

### Evaluation focus and dimensions

The focus of the evaluation was on the outcome of the CBMP. Therefore, the evaluation used relevance, effectiveness, and impact dimensions from the Organization for Economic Cooperation and Development (OECD) evaluation framework [19]. The OECD evaluation framework has five dimensions (Relevance, Effectiveness, Efficiency, Impact, and Sustainability). However, the efficiency and sustainability dimensions were not included in this study.

The efficiency dimension was not applicable to measure in this program because of two main reseasons. First, during the evaluability assessment, the program has no separate financial documentation for each healthcare organization. Secondly, according to the definition of efficiency in the OECD framework, there should be a similar program to assess efficiency [19]. Unfortunately, there is no comparable program to perform the program’s cost-effectiveness in Ethiopia. Furthermore, regarding sustainability, the program only implemented for two years, which is not appropriate to assess the sustainability of the study at this stage.

### Sample size and sampling procedure

A total of 40 health facilities (4 hospitals and 36 health) were taken from the project implementation and the comparison sites. The sample was taken based on the WHO recommendation for Assessing the Operationality of District Health Systems [20],

All health facilities (primary hospitals and health centres) supported by the CBMP in the Amhara National Regional State were included in this evaluation. The program was implemented in three zones in the Amhara region (East Gojjam, South Wollo, and Central Gondar zone. The program selected one district from East Gojjam and South Wollo zones each and two districts from the central Gondar zone.

Sample size for the comparison was selected within the same zone for each facility to match with the intervention sites. One district was randomly selected from the same administrative zones that the intervention district was found by excluding the intervention district. A total of 17 health facilities were selected for the comparison group. Key informant interviews were conducted to measure relevance dimension, and multiple purposive sampling techniques were used to select the informants. A total of 24 key informant interviews with health facility managers and HIT officers were conducted from the four intervention districts.

### Operational definitions

Relevance was used to assess the appropriateness of the program’s activities to the health facilities (Hospital/ Health center) that the program was implemented. The dimension was evaluated qualitatively by interviewing the health institutions head and HMIS officers about the importance of the CBMP and the need for health data quality intervention on their institution [19].

Effectiveness was measured by the extent of CBMP objectives achievements. It was measured by comparing the program target objective with its current data quality level of the health institutions [18].

Impact: This evaluation dimension assesses the contribution of CBMP on data quality. This dimension was assessed by taking a comparison group and identifying the difference between the intervention and comparison groups [19].

Data quality was measured using nine indicators with a sub-dimension of accuracy, completeness, and timeliness subdimensions [21]. According to the Ethiopian HIS management, there are more than 221 reportable data elements which consists both services delivery and diseases specific.

Data accuracy: The data accuracy was measured using a proxy indicator of conducting the required Lot Quality Assurance Sampling (LQAS), as stated by the ministry of health checklist [21]. It shows data compiled in reporting forms are accurate and reflect consistency between what was in reporting forms and what is in registries at the health facility level [22]. Then 12 data elements from the total reportable are taken randomly and then check whether the reported was consistent with the registered. Accordingly, we counted the total yes out of 12 and the decision was given from 0 to 100 % to see the level of accuracy.

Data completeness: The completeness of the data is assessed by measuring whether all the entities which are supposed to report do so [1]. Five indicators were used to assess the data completeness sub-dimension with content and representative completeness.

Report timeline: Timeliness of data was assessed by measuring whether the entities which submitted reports did so as per the national schedule. The national schedule expects to receive reports from health posts between the 21st and 23rd days of the month according to the Ethiopian calendar. Then health center are expected to submit a report to woreda between the 24th -26th days of the month [1]. A total of three indicators are used to measure the timeliness sub-dimension of data quality.

### Data collection tools and procedures

For the measurements of relevance, a semi-structured questionnaire was used to interview the health facility managers/heads and HMIS officers. The questionnaires were developed from guidelines and a study conducted on OECD/DAC criteria for international development evaluations [19]. The proving questions and the interview were conducted using the local language (Amharic). The head and HMIS officers of the selected health facilities were interviewed through probing questions about the relevance of the CBMP for the health institutions. To ensure the data quality, the interview was conducted by a BSc graduate with public health and had experience in HMIS and have a prolonged engagement on the intervention site were used. Also, the conversation was conducted with the local language (Amharic). Moreover, a field note was taken, and the interview was recorded for further analysis.

A structured questionnaire was used for the measurements of effectiveness and impact dimensions. The questionnaire is a standardized, valid tool with a Cronbach alpha value of 0.7 were used by the Ministry of Health – Ethiopia to assess the health facility with structure, data quality, and information use dimension [21].

The quantitative data were collected at the intervention and comparison group using a structured questioner. A similar tool was used to measure the data quality of the intervention and comparison groups. The availability of requested data was verified from the previous report, PMT logbook, minute, supportive supervision checklist, and observation.

The quantitative data were collected first and followed by qualitative data. The key informant interview and structured questioners were used.

To assure the data quality, two-day training was provided for six data collectors and three supervisors on the objectives of the evaluation, procedures, and overall formats to familiarize them with the tool. The data collectors were BSc graduates with a health background and had experience in the HMIS Besides; the data collectors have prolonged contact with the head of the health facility and HMIS officers. Moreover, a field-note was taken while recording the data. The interview was conducted with the local language (Amharic) then transcribed and translated for analysis. The principal investigator and supervisors were supervised the data collection process, overall completeness of the questionnaires and accuracy daily.

### Data management and analysis

Quantitative data were entered into Epi-Data version 3.1 software and transferred to STATA version 14 for analysis. For qualitative data, field notes were written as fair notes. Also, the key-informant interview was recorded and transferred to the computer for analysis. The qualitative and quantitative data were analyzed separately. However, the data were mixed in the interpretation phase. Though the result is dominantly quantitative, the qualitative data was used to complement the study.

The change in data quality components was calculated using paired t-test to assess the significant mean data quality difference between the CBMP intervention site and comparison health facilities. First, the impact of the CBMP was analyzed using a propensity score matching (PSM) technique. Then the average treatment effect was estimated to assess the contribution of CBMP on data quality.

Finally, the qualitative data were transcribed and translated for analysis and thematic content analysis was done.

#### Judgment matrix

The judgment criteria’s for data quality were adopted from evaluations entitled “assessing the ability of health information systems in hospitals to support evidence-informed decisions in Kenya” and “assessment of data quality and information use of the community health information system: a case study of karurumo community health unit-embu county” [23, 24]. The impact judgment criteria were determined and agreed upon with stockholders (Table 1).

**Table 1:** The judgment matrix of CBMP in selected health facilities in Amhara National Regional State, Ethiopia, 2020

Dimensions	Score	Judgment
Effectiveness	90% and above	Excellent
	80%– 90%	Very Good
	70%– 80%	Good
	60%– 70%	Fair
	Less than 60%	Poor
Impact	ATE ≥ 25 = Agreed Impact = ATET/25*100	> 90% = Excellent 80% - 90%= Very Good 70%-80% = Good 60%-70% = Fair < 60 %= Poor
Overall Outcome	(Effectiveness + Impact) /2	> 90% = Highly satisfactory 75%-90% = Satisfactory 50%-75% = Unsatisfactory < 50 %= Highly unsatisfactory

*ATET* Average treatment effect on the treated

## Results

### Description of study participants and facilities

A total of 40 health facilities (23 interventions sites and 17 comparisons) (Table 2) were included in this evaluation to measure the relevance, effectiveness, and impact of CBMP. Moreover, twelve health facility heads and HIS officers were interviewed from the CBMP health facilities in this evaluation.

**Table 2:** Distribution of health facilities included in the evaluation of CBMP, Amhara region, 2020

Zones	Interventions		Comparisons		Total
	HC	Hospital	HC	Hospital	
East Gojjam	6	1	6	1	12
South Wollo	5	0	4	0	9
Central Gondar	10	1	5	1	17

*HC *Health Center*; CBMP *Facilities having; *CBMP Comparison = facilities without CBMP intervention*

### HIS infrastructure

Among the 40 facilities, 35 (87.5 %) have electronically assisted medical record unit; 34 (85 %) of health facilities had a functional computer dedicated to DHIS 2. Twenty-two of the intervention health facilities had a functional computer with DHIS 2 system. Among the 17 facilities in the comparison group, 6 (35.29 %) properly filled individual medical card and easily accessible for clients. In the intervention group, 14 (60.86 %) facilities filled individual medical card properly (Table 3).

**Table 3:** HIS inputs for health facilities with CBMP and comparison group, Amhara National Regional State, Ethiopia, 2020

Variables		Category	Intervention n (%)	Comparison\ n (%)
Medical Record Office	Standard medical record unit	Yes	14 (60.86)	6 (35.29)
		No	9 (39.13)	11 (64.70)
	Standard Shelves	Yes	16 (69.56)	4 (23.52)
		No	7 (30.43)	13 (76.47)
HMIS Unit	Dedicated desk (Office)	Yes	22 (95.65)	9 (52.94)
		No	1 (4.54)	8 (47.05)
	Functional Computer of DHIS 2	Yes	22 (95.65)	12 (70.58)
		No	1 (4.54)	5 (29.41)
	Staff for HIS implementation	Yes	19 (82.60)	12 (70.58)
		No	4 (17.39)	5 (29.41)

*HMIS* Health Management Information System; Intervention: *CBMP; NCoD* National Classification of Disease; Comparison: health facilities without CBMP intervention

Nearly two-thirds of the project sites’ health facilities had no shortage of individual medical cards, registers, and tally sheets in the past six months. However, 76.47 % of the health facilities in the non-project sites run out of medical cards, registers, or tally sheets in the past six months.

**Fig. 4:**
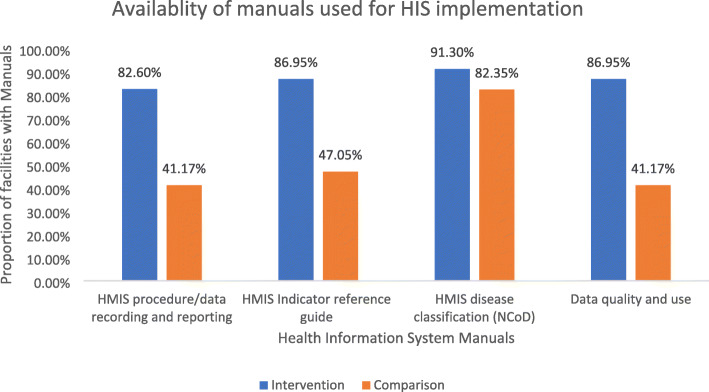
Availability of manuals in CBMP health facilities and the comparison group in Amhara National Regional State, 2020

Facilities that received CBMP have a higher proportion of the availability of manuals needed for the successful implementation of the health information system. The range varies from 91.3 % (HMIS disease classification) to 82.6 % of (HMIS procedure/ data recording and reporting) for the intervention health facilities. The value is much lower in the case of comparison health facilities, which varies from 82.35 % (HMIS disease classification) to 41.17 % (HMIS procedure/ data recording and reporting manual and Data quality and use) (Fig. 4).

### Prosecution of the HIS

About 22 (95.65 %) of facilities in the intervention group and 11(64.70 %) of facilities in the comparison group use checklist during the supervision.

Health information system capacity need assessment was not conducted for 82.4 % of the comparison group’s health facilities. In more than half (65.2 %) of the facilities under the CBMP conducted HIS need assessment in the past six months. Among 15 health facilities, HIS need assessment was conducted and of these 9 of them communicated to the next level about the finding and need.

### Relevance of CBMP

Data from the recordings and field-notes were thematized into four themes; (1) Prior data quality problems before the implementation of the CBMP, (2) Perceived contribution of the program to the data quality improvement, (3) The readiness of the health facilities to maintain the current data quality without the support of the CBMP and (4) Strength and weaknesses of the CBMP implementation.

All participants believed that there was a data quality problem in their health facilities before the CBMP implementation. The problems include discrepancies between tally, register, and report, lack of documentation for conducted activities. Moreover, the inappropriate use of register was some of the problems that the respondents mentioned. This was described by the participant as follow:

“*In regards to data quality, there were many problems in our health institution before CBMP implementation. For example, discrepancies between tally and register. Secondly, the lack of documentation for activities conducted in the health facility.*“

(A facility head, Awobal district)

“*There was a data quality gap in our facility before the program implementation. The gaps were the result of lack of attention from the clinical staff which results in fallacy in tally and register*. In addition, incomplete data was a major problem.“

(HIT officer, Central Gondar district)

In addition to the CBMP, there are internal and external efforts to increase data quality in the health facilities. The respondents mentioned that there was a performance monitoring team, the quality improvement team, and supportive supervision from the district health office. A typical response from the health information system officer at the Tehuledere district:

“*Yes…there are activities to improve data quality in our facility. These are the performance monitoring team and a quality improvement project. In addition, we got support from the CBMP for two years and are still working with us*.“

(HIT officer, Tehuledere district)

Moreover, participants thought that the mentors know data quality and health systems. The majority of the respondents said the number of mentors was enough for the program activities. However, some respondents believed that the activities are linked to all the health systems that the allocated number of mentors is not sufficient leads to much burden for the mentors.

All of the respondents believed that the CBMP contributed to the data quality improvement in their facility. They think that the program contributed to data quality improvement through changing the health facility staff’s attitude towards the importance of quality data. Also, the program gave training on data quality for the staff.

“*Definitely…In the previous practice, there were a lot of gaps in data quality and information utilization. Our baseline data before the implementation of the program was around 41 %, but after the CBMP, we managed to achieve around 91 %.*“

(Facility head, Tehuledere district)

“*Yes… in our health center, the CBMP has a very crucial role. As I said before, there was low data quality in our facility, but after getting the mentorship, even though we did not solve all the problems, there is a huge improvement in data quality in our day-to-day work. The program helps us to generate and disseminate quality data.*“

(Facility head, Awobal district)

“*I got many things from the mentorship for my future carrier. In the past, the staff in our facility did not use data for decision making, but the CBMP program identifies the skill gaps particularly for the HIT. We received maintenance training at Debre Markos. In addition, we got training on data quality, information used, and infrastructure to build a database in our facility*.“

(HIT, Awobal district)

Most of the participants responded that they could maintain the existing data quality without additional support. They argued that the health facility staff has a better understanding of the data quality and knowledge to record data completely and accurately. However, some respondents doubt to maintain the current data quality. They mentioned that some of the issues are still difficult to fulfill with the health center capacity. They stated that they could not afford to print the register if it is run out. Moreover, the staff shift from the facility who does not get training may affect the current data quality. However, they declared their commitment regarding technical issues that could be performed with their ability.

The participants in the study indorse things to improve in the implementation of CBMPs. The first thing was that the mentorship was not conducted regularly, that it needs improvement in keeping the timeline of supporting every quarter. Some interviewees also recommend full staff training on data quality, i.e., health facility staff work on different departments through rotation and delegation.

“*The endorsement I have for the program is about the schedule. Sometimes the mentors do not come with the timeline of every quarter. I recommend keeping the schedule within a week or two after completion of the quarter*.“

(HIT, Awobal district)

In conclusion, the program is needed by the health facilities to improve the data quality and information utilization gaps. The program gives training and mentorship to improves the data quality and information utilization culture in the organization.

### Effectiveness of CBMP

In this evaluation, 23 health facilities that received CBMP were used to measure the effectiveness of CBMP on improving health data quality. A total of 9 indicators were used to measure the data quality with the data accuracy, completeness, and timeliness dimensions.

The average data quality of health facilities that received CBMP was 89.06 (95 % CI: 84.23, 93.88. The CBMP is statistically effective in achieving the data quality change in the pilot health facilities. However, the overall judgment based on the indicator showed that the program has good performance on effectiveness (Table 4).

**Table 4:** Summary of indicators to measure data quality in the CBMP, Amhara National Regional State, 2020

Sub-dimensions	Indicators	Weight (W)	Score (S)	Percentage (S/W*100)	Judgment parameter
Accuracy	Proportion of lot quality assurance sampling (LQAS) conducted	10	8.17	81.73	Very Good
Completeness	Availability of log book to track completeness of report	2	2	100	Excellent
	The proportion of report completeness in the past six months	4	3.65	91.25	Excellent
	Complete report submitted to the highest level	1.5	1.43	95.3	Excellent
	Complete report received from the lowest level	1.5	1.23	82.6	Very Good
	The proportion of content completeness in the previous six months	3	2.74	91.3	Excellent
Timeliness	Availability of logbook to track the timeliness	1	1	100	Excellent
	The proportion of report received from the lower level by the national schedule	4	3.74	93.21	Excellent
	The proportion of report submitted to the next level by the national schedule	3	2.74	91.30	Excellent
Overall		30	26.72	89.06	Very Good

Judgment: > 90% = Excellent, 80% -90%= Very Good, 70%-80% = Good, 60%-70% = Fair, < 60 %= Poor

### Impact of CBMP

**Fig. 5:**
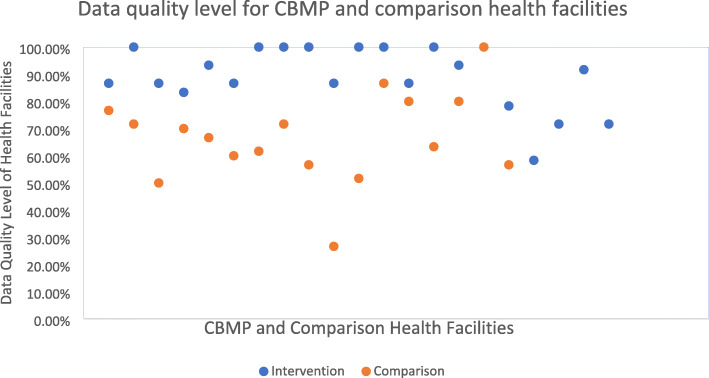
Data quality level of CBMP and comparison health facilities in Amhara National Regional State, 2020

The comparison and CBMP health facilities’ average data quality was 66.5 % (95 % CI: 57.9–75) and 89.1 % (95 % CI: 84.2–93.9), respectively. The data quality was measured for each health facility (Fig. 5).

The matching characteristics (facility type, catchment population, catchment institution number and standard medical unit) for the propensity score matching is summarised in Table 5.

**Table 5:** summary of the matching characteristics with the comparison and intervention group, Amhara National Regional State, 2020

Variable		Obs	Mean	Std.dev	Min	Max
Comparison	**Data quality**	17	66.47	16.60	26.67	100
	**Facility type**	17	1.12	0.33	-	-
	**Catchment population**	17	36082.29	13133.65	16365	58709
	**Catchment Institution**	17	5.12	2.06	3	11
	**Standard Medical Unit**	17	0.35	.49	-	-
Intervention	**Data quality**	23	89.06	11.16	58.33	100
	**Facility type**	23	1.09	0.29	-	-
	**Catchment population**	23	31204.43	21984.36	10676	106167
	**Catchment Institution**	23	4.78	2.26	2	12
	**Standard Medical Unite**	23	0.61	0.50	-	-

*Obs* Observation; *Std.dev* Standard Deviation; *Min* Minimum; *Max* Maximum

Based on the Probit regression, the average treatment effect is estimated using different matching methods. On the nearest neighbor methods, being in the treated group increase the data quality by 27.75 % points (95 %CI: 17.94, 37.58) (Table 6).

**Table 6:** The average treatment effect on the treated estimation of CBMP on data quality using different methods in Amhara National Regional State, Ethiopia

Estimation Methods	No. treated	No. comparison	ATET	Std. Err	95 % CI
T-test	23	17	22.59*	4.39	13.71, 31.47
Nearest neighbor	23	11	27.75*	5.00	17.94, 37.57
Radius	22	17	23.72*	4.87	14.38, 33.07
Kernel	23	17	26.5*	4.58	17.52, 35.5
Stratification	23	17	24.02*	4.96	

* Probability of t less than 0.05; *No* Number; *ATET* Average Treatment Effect on the Treated; *Std.Err* Standard Error; *CI* Confidence Interval

The program has excellent (92.5 %) performance on the impact dimension based on the judgment criteria. In summary, the weight given for the dimension of outcome evaluation by effectiveness and impact, the overall score is 90.75 per cent that the program. Thus, the program achieves its objective in a highly satisfactory condition (Table 7).

**Table 7:** Summary of the overall performance of the CBMP on data quality at health facilities in Amhara National Regional State, 2020

Dimensions	Relative weight	Score	Achievement (S/W*100)	Judgment
Effectiveness	50	44.5	89 %	Very Good
Impact	50	46.66	92.5 %	Excellent
The overall outcome of CBMP			90.75 %	Highly Satisfactory

Judgment for Effectiveness and Impact: > 90 % = Excellent, 80 % -90 %= Very Good, 70 %-80 % = Good, 60 %-70 % = Fair, < 60 %= Poor

Overall Outcome of CBMP Judgement: > 90 % = Highly Satisfactory, 75 %-90 % = Satisfactory, 50 %-75 % = Unsatisfactory, < 50 %= Highly unsatisfactory

The overall judgment criteria were adopted from the literature on “evaluating development operations: methods for judging outcomes and impacts,“ which is published by world bank [25].

## Discussion

In this evaluation, the OECD criteria were used to estimate the effectiveness, relevance and impact of CBMP on data quality. The relevance dimension was used to describe the importance of the program and possible implementation and challenges to avoid “black box” evaluation. The program performs 89 % and 92.5 % on effectiveness and impact dimensions based on the judgement matrix. The overall outcome of the CBMP program was 90.75 %. In this regard, the judgment criteria of the program were found to be highly satisfactory.

The evaluation findings showed that there was a data quality problem in the health facilities before the implementation of the CBMP. Some of the issues were discrepancies between tally, register, and report, lack of documentation for conducted activities, and inappropriate use of register in the facilities. The findings are supported by the evidence from the Ethiopian institute of public health data quality review report, which states that there were problems in data compilation, guidelines, and reporting, and also there is internal inconsistency [7]. Correspondingly, it is supported by the WHO that declares that all data in health care has particular limitations in terms of measurement error, missing values, and human mistakes in data entry and analysis [1]. Furthermore, the finding is in line with that of the baseline assessment done by the CBMP. It showed that there were gaps in health data documentation, analysis, and utilization of data by the health facilities [14]. Low data quality results in low utilization and, if it is used, wrong evidence-based decision making.

Different arrangements were available in the health facility to increase data quality and information utilization to support the health information system. These are a performance monitoring team, quality improvement team, and supportive supervision from the district health office. Even if the structure of the system exists, there was a gap in implementation. The low performance in data quality despite the existence of the structure is a piece of clear evidence for a gap. This result is supported by a study conducted developing countries on the traditional inspection and control methods reviled that it could lead to a limited performance in supportive supervision and demoralize the staffs [26].

The data quality on the CBMP intervention health facilities was 89.06 %, which makes the program statistically effective in achieving its objective data quality of 90 %. The qualitative study could explain this presented that the mentors knew about the data quality and health systems. Furthermore, as described by the head of the facilities and HMIS officers, the mentors’ commitment could result in the program’s achievement to its target. The fact that the mentors are from the academic institution who had experience teaching could help the program achieve its objectives. The program’s effectiveness is supported by a systematic review of the effectiveness of capacity building interventions relevant to public health revealed that capacity-building activities enhance knowledge, skill, and confidence that leads to behavioural change at a system level [27].

The proportion of LQAS conducted and received of complete reports from the lower health facility indicators for the data quality needs attention in the implementation of the program. This will result in low data quality in the health facilities. As explained by the result from the interview with the facility head and HMIS officers, the reasons could be the inconsistent timeline of mentorship of CBMP and lack of training for all the staff members.

The average treatment effect on the treatment of the CBMP on data quality was 27.75 % points, which means the program increases the data quality by 27.8 % points. The finding is supported by the qualitative analysis of the head of health facilities and health information system officers. Respondents believed that the program helps them to increase the data quality in their facility. It contributed by changing the health facility staff’s attitude towards the importance of data quality. Likewise, the program gives training on data quality for the staff as well. This is also supported by the statistically significant mean difference in data quality between the intervention and comparison of health facilities. Moreover, the finding is supported by a case study conducted in Ethiopia on supportive supervision revealed that it has a significant contribution to data quality [28].

### Limitation of the evaluation

This evaluation has a limitation of selection bias because of the lack of randomization in the process. This may overestimate or underestimate the result. All the intervention groups were included in the evaluation to minimize the selection bias, and the comparison district was selected using simple random sampling. In addition, PSM was conducted to minimize selection bias while estimating the average treatment effect on the treated.

Moreover, the lack of baseline data for the comparison group makes it is difficult to conclude that the program has the exact attribution for the observed change in data quality. However, the study assessed the outcome difference between the intervention and comparison of health institutions by matching the baseline characteristics as the intervention group.

## Conclusions

In conclusion, the outcome of the CBMP was highly satisfactory, based on the judging criteria. The program was effective and contributed to the data quality improvement in the pilot health facilities. Moreover, it was needed by the health facilities to improve the data quality gaps in the health facilities.

Regular mentorship and provision of training for all healthcare workers about data quality were suggested by the head of health facility and health management information system officers for the program implementers.

## Data Availability

The datasets used and/or analyzed during the current study are available from the corresponding author on reasonable request.

## Abbreviations

ATE: Average Treatment Effect
CBMP: Capacity Building and Mentorship and Partnership
CI: Confidence Interval
Coef: Coefficient
DHIS2: District Health Information System two
HIS: Health Information System
HMIS: Health Management Information System
LQAS: Lot Quality Assurance Sampling
OECD: Organization for Economic Cooperation and Development
PSM: Propensity Score Matching
Std. Err: Standard Error
WHO: World Health Organization
